# The Complex Mechanism of the *Salmonella typhi* Biofilm Formation That Facilitates Pathogenicity: A Review

**DOI:** 10.3390/ijms23126462

**Published:** 2022-06-09

**Authors:** Fahmida Jahan, Suresh V. Chinni, Sumitha Samuggam, Lebaka Veeranjaneya Reddy, Maheswaran Solayappan, Lee Su Yin

**Affiliations:** 1 Department of Biotechnology, Faculty of Applied Sciences, AIMST University, Bedong 08100, Kedah, Malaysia; fahmidabristy.btge@gmail.com (F.J.); sumithasamuggam@gmail.com (S.S.); maheswaran_s@aimst.edu.my (M.S.); 2 Biochemistry Unit, Faculty of Medicine, Bioscience, and Nursing, MAHSA University, Jenjarom 42610, Selangor, Malaysia; 3 Department of Microbiology, Yogi Vemana University, Kadapa 516005, Andhra Pradesh, India; lvereddy@gmail.com

**Keywords:** *Salmonella typhi*, typhoid, gallbladder, biofilm

## Abstract

*Salmonella enterica* serovar Typhi (*S. typhi*) is an intracellular pathogen belonging to the Enterobacteriaceae family, where biofilm (aggregation and colonization of cells) formation is one of their advantageous traits. *Salmonella* typhi is the causative agent of typhoid fever in the human body and is exceptionally host specific. It is transmitted through the fecal–oral route by consuming contaminated food or water. This subspecies is quite intelligent to evade the innate detection and immune response of the host body, leading to systemic dissemination. Consequently, during the period of illness, the gallbladder becomes a harbor and may develop antibiotic resistance. Afterwards, they start contributing to the continuous damage of epithelium cells and make the host asymptomatic and potential carriers of this pathogen for an extended period. Statistically, almost 5% of infected people with *Salmonella typhi* become chronic carriers and are ready to contribute to future transmission by biofilm formation. Biofilm development is already recognized to link with pathogenicity and plays a crucial role in persistency within the human body. This review seeks to discuss some of the crucial factors related to biofilm development and its mechanism of interaction causing pathogenicity. Understanding the connections between these things will open up a new avenue for finding therapeutic approaches to combat pathogenicity.

## 1. Introduction

The World Health Organization estimates that 11–20 million cases of typhoid fever occur worldwide each year, resulting in approximately 150,000 fatalities. Although typhoid fever is widespread across different countries, it is more frequently found in Bangladesh, China, India, Indonesia, Laos, Nepal, Pakistan, and Vietnam, accounting for approximately 80% of cases. It is more prevalent among poor people and endangered groups where the populations lack access to safe drinking water and proper sanitation. Typhoid fever is an acute sickness characterized by high fever, lethargy, and stomach discomfort that comes from the extra-intestinal compartment invasion even without the development of any inflammation or diarrhea [1]. Once ingested, it passes the intestinal barrier via microfold cells and causes invasion of the mucous membrane of intestinal cells. It can also infiltrate the macrophages and begin replication within them. Then, with the help of the macrophages, this pathogen moves into the liver, pancreas, and spleen, where the liver starts shedding into the gallbladder. Due to uncontrolled antibiotic drug usage, the expansion of multidrug-resistant *S. typhi* has increased typhoid fever recurrence rates during the last decade, which has added to the burden of the disease [2,3]. Almost every first-line antibiotic has been found to be ineffective, and up to 60% of all isolates have multidrug resistance [3]. Usually, bacteria can grow in two ways: as single planktonic cells or as a community within a biofilm [4]. Biofilms are aggregates of cells attached to the surface encased in an extracellular polymeric substance (EPS). Biofilm formation is a method of communication among many microbes. At the same time, biofilm also protects them from certain environmental stresses, such as osmotic shifts, oxidative stress, metal toxicity, dehydration, radiation, host immunity, antimicrobial agents, and disinfectants [5,6,7,8,9,10]. A special feature of this biofilm is that it allows the bacteria to strategize remarkably to coordinate functions and develop complex behaviors that will be advantages for its virulence on the host. Biofilms are the most common bacterial growth mechanism, accounting for roughly 80% of all bacterial infections [2,9]. The hosts’ innate and adaptive immune responses may be insufficient to eradicate the pathogen within the well-established biofilm [11,12]. The infection caused by S. *typhi* is often related to high levels of replication and concomitant burden of the pathogen through the formation of biofilm that alters the bacterial growth physiology, which in turn allows for high levels of antibiotic administration. As a consequence, it also develops a tolerance to other host responses, such as the complement system, antimicrobial peptides, antibodies, and phagocytic activity by neutrophils and macrophages [13] Moreover, the biofilm formation also leads to the shedding of planktonic bacteria and permits the entry of biologically active molecules into the host that causes phenotypic effects on the host immune state and is important for maintaining a favorable niche by altering the activation of innate immune receptors, inhibiting apoptosis, inducing an inappropriate immune response, or causing immunosuppression [13,14]. Though the ileum, liver, spleen, bone marrow, and gallbladder are the most prevalent infection sites, the bile-rich gallbladder is the leading site of human serovar Typhi transmission. Like other enteric infections, Salmonellae are highly resistant to bile, and respond by up-regulating resistance-related genes [15]. Chronic *S*. *typhi* colonization cannot be resolved with antibiotics; gallbladder resection is the only option. However, further infection foci can form in the bile duct, mesenteric lymph nodes, or liver [16,17]. *Salmonella typhi* infections can last for decades, while infected people are highly contagious and often asymptomatic, which complicates the identification of carriers. Though the molecular mechanism of its survival in the host and its pathogenic properties are poorly understood, some important factors have been reported to be associated with its pathogenicity. Because of it being highly host specific, there is little information regarding *S.* typhi interaction with the gallbladder. That is why pathogenesis of *S. typhi* in vivo studies usually uses a mouse model infected by *Salmonella enterica* serovar Typhimurium (*S. typhimurium*). *Salmonella typhi* shares 80% of its genomic sequence with *S. typhimurium,* yet the pathogenicity of these two strains is vastly different [18,19]; *S. typhi* causes typhoid fever in humans, while *Typhimurium* causes gastroenteritis in humans. Compared with planktonic cells, *S. typhimurium* biofilms grown on microplates are up to 2000-fold more resistant to ciprofloxacin [20]. Salmonella biofilms developing on gallstones are thought to be the source of this antibiotic resistance and longtime persistence. Studies in *S. typhi* endemic regions, including Chile, Bolivia, Ecuador, India, Pakistan, Japan, and Korea, have found that over 90% of chronically infected carriers have gallstones, which is a major predisposing factor for gallbladder cancer [21,22,23]. Though this carcinoma has been linked to genetics and lifestyle, the most prominent risk factors include *S. typhi* infection and gallstone disease [24]. Furthermore, other serious typhoid complications are typhoid intestinal perforation (TIP), gastrointestinal hemorrhage, hepatitis, myocarditis, shock, encephalopathy, pneumonia, and anemia [15,25]. Millions of people worldwide contract typhoid every year, despite extensive treatment and preventative efforts. Due to all these adverse impacts on human health and the high frequency of typhoid fever in many areas around the world, it becomes urgent to comprehend the strategies involved in the transmission and survival of *S. typhi*.

## 2. Environmental Factors Associated with Biofilm Development

### 2.1. Bile Mediated

**Figure 1:**
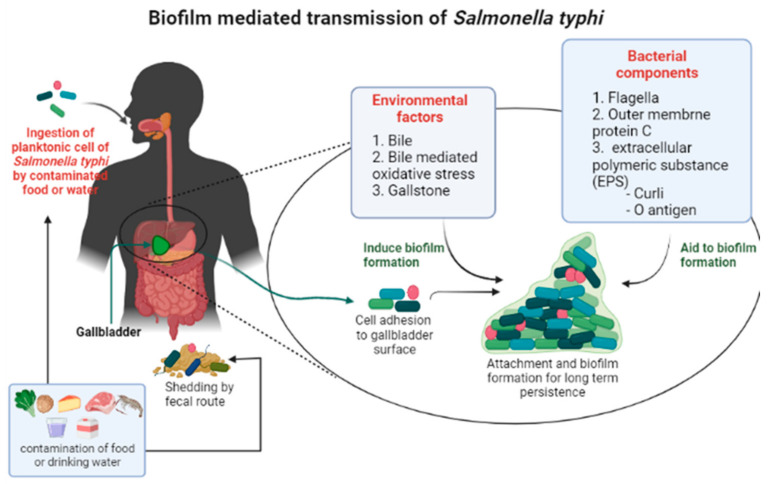
A schematic view of *Salmonella typhi* transmission process facilitated by biofilm formation in the gallbladder.

To survive within the human body for an extended period, *S. typhi* utilize the gallbladder environment. The pathogen enters into the gallbladder using the vasculature or the bile ducts, which originate in the liver. Henceforth, bacteria are ready to shed from the biofilm and disseminate into the environment for further spreading through feces and urine (Figure 1) [26]. Bile acid, a digestive fluid produced in the liver, is stored in the gallbladder, and partially works as a reflex to enteric infection by large intestine bacteria [27]. Bile is extremely toxic to microorganisms that are not adapted to intestinal conditions. Bile and other antibiotics can kill bacteria, but they can also make the bacteria more resistant to them by the formation of persister cells [26]. This non-inherited and epigenetically modified strain make them more resistant to many drugs and more capable of making infections that last a long time. Reports say that *S*. *typhi* also forms persister cells when it is treated with ciprofloxacin and ampicillin [28,29]. Increasing shreds of evidence showed that the bile is linked to a variety of phenomena within the cell, including the induction of oxidative stress, DNA repair mechanisms, sugar metabolism changes, and protein misfolding [30]. Bile also influences biofilm formation in many pathogenic bacteria and some indigenous commensals [30]. However, some enteric bacteria, such as typhi, must have developed unique defense mechanisms to counteract the harmful effects of bile. There is evidence that bile salts penetrate bacterial cells and control numerous gene loci involved in oxidative stress, cell and membrane protein production, efflux systems, and other survival processes [8,27,30,31].

Bile is a significant regulator of gene expression in Salmonella, affecting 10% of the genome, including virulence, motility, and metabolic genes [32]. Since *S*. *typhi* is highly host specific, humans are the only carrier of this infection. Specifically, biofilm development inside the bile-salt-enriched gallbladder can aid the pathogen’s persistence. To counteract this bile-salt-mediated stress and protect themselves against oxidative damage, some bacteria have been shown to increase the endogenous production of anti-oxidative enzymes, primarily superoxide dismutase (SOD) and catalase [33,34,35]. This mechanism also holds true for S. *typhi*. It has been proved that, during oxidative stress, *S. typhi*’s quorum sensing (QS) controls the amount of these enzymes by up-regulating their expression [29]. Thus, it can be said that environmental stress contributes to biofilm formation.

### 2.2. Gallstone Mediated

Though the exact mechanism of biofilm formation is unknown, it has been established that any abnormalities or infection of the gallbladder facilitates the long-term asymptomatic carriage of *S. typhi* [22,36,37]. Any inflammation in the gallbladder or bile ducts is referred to as cholelithiasis that can be caused by gallstones, and it is one of the most common medical diseases that necessitate surgery [38]. It is reported that approximately 80% of chronic carriers of *S. typhi* have gallstones, and salmonella carriers who also have gallstones are 8.47 times more likely to develop gallbladder cancer [22,39,40,41]. Environmental stress and genetic manipulation of the host body concomitantly contribute to the development of cholelithiasis [37,42]. The pathological aspects of *S*. *typhimurium* infection in mice are comparable to those of *S. typhi* infection in humans [43]. In order to test the concept, a murine model has been developed, in which mice were fed a cholesterol-inducing lithogenic diet. According to this study’s findings, gallstone-forming animals are more susceptible to biofilm development than control mice when infected with serovar Typhimurium [44]. Bile and gallstones work together to promote biofilm growth, either using signaling molecules or providing a niche for gallstone formation [45]. To support this hypothesis, researchers cultured bacteria in Luria Bertani (LB) broth, and LB broth with 3% bile, followed by incubations for the next seven days. After that, it was incubated for four days with gallstones. Surprisingly, the results revealed that bacteria only successfully produced complete biofilm when combined with bile and gallstones, but not when cultured alone [45]. *Salmonella enterica* produced biofilms poorly on calcium bilirubinate (another type of gallstone) compared with cholesterol in a tube biofilm assay, confirming that *S. typhi* has a particular binding affinity for cholesterol gallstones [44].

## 3. Bacterial Components That Aid in Biofilm Formation

Several factors associated with bacterial biofilm were investigated to find crucial factors for the formation of mature *S. enterica* biofilms on the surface and the cholesterol-coated surfaces of gallstones. Among them, flagella and fimbriae have been proved as crucial biofilm initiation factors for numerous bacteria (*Pseudomonas aeruginosa*, *Escherichia coli*, etc.) in the environment [45,46,47]. Flagella have been found to play a vital role in the production of biofilms, particularly in the early phases when microcolonies are forming. Additionally, flagella are required for bacterial movement to the surface for attachment and for the propulsion of the organisms in search of other bacteria [46,47,48]. Moreover, a reduction in serovar Typhi flagellar expression leads to lower inflammation [49]. A mutant that was deficient in flagellar development (non-motile) was investigated to see if flagella could influence the biofilm formation by Salmonella on gallstones; a modest biofilm formed after 14 days (approximately 2 weeks), although the phenotypic attributes were quite different from the *S. enterica* serovar Typhimurium wild type [45]. Moreover, EPS was also not found with the non-motile bacteria on the gallstone. From this, it can be concluded that Salmonella flagella may be involved in EPS secretion, as well as early adhesion and microcolony development on gallstones [45]. The tube biofilm assay (TBA) was designed to research biofilm development on cholesterol that worked as an in vitro surrogate for gallstones 50. In the TBA, a pool of transposon mutants was tested with a daily passage of planktonic (non-adherent) bacteria [36]. Using this approach, researchers have discovered that the flagellin subunit (FliC) is essential for early cholesterol-coated surface attachment, and the loss of outer-membrane protein C (OmpC) impeded cholesterol binding and biofilm formation [44]. In addition, *S. typhi* outer membrane proteins (Omps) are powerful immunogens that induce long-lasting and protective immunity.

Further research revealed that the hyper-fimbriate phenotype had a deleterious impact on the early phases of biofilm development on cholesterol [44]. Thus, in *S. enterica*, the first attachment phase of biofilm formation may entail a combination of flagella and outer-membrane proteins, which can be concealed by the over-expression of surface fimbriae [50]. However, the broad function of flagella in the production of serovar Typhi biofilms is yet to be fully explored. 

A bile-induced extracellular polymeric substance (EPS) is required to produce biofilms on cholesterol-coated surfaces (gallstones) and cell-to-cell interaction [6,36]. Several components of EPS have been found in Salmonella-species biofilms. These include cellulose, colanic acid, the Vi antigen, curli fimbriae, the O antigen capsule, and some biofilm-associated proteins [44,45,51,52]. Though the Vi antigen has not been shown to affect biofilm formation in *S. typhi*, the O-ag is required for Salmonella biofilm formation on the cholesterol-coated surface in serovar Typhimurium, Typhi, and Enteritidis, and Curli is the most crucial contributor to biofilm development [36,45]. Surprisingly, Curli-deficient mutants have been reported to have a considerable decrease of 45% in the biofilm compared with wild type [53]. Bile has been demonstrated to affect EPS synthesis and O-ag capsule induction; it has been reported that wild-type serovar Typhimurium, serovar Typhi, and serovar Enteritidis growth, in 3% bile, improved O-ag capsule induction [30,36].

## 4. Genes and Regulatory Molecules Involved in Biofilm Formation

A vast and intricate regulatory network governs bacterial biofilm formation. Many genes are associated with this entire system (Table 1). Several non-coding RNA and regulatory molecules have also been discovered to play essential roles in this system (Table 1). There are, however, many more to be explored.

### 4.1. Salmonella Pathogenic Islands (SPIs) 

Salmonella pathogenesis-related virulence genes are mostly found on chromosomes and plasmids. These virulence-associated genes and regulators are found in specific chromosomal regions, known as Salmonella pathogenic islands (SPIs), and *typhi* has been identified with ten SPIs [19]. Among them, SPI-1 and SPI-2 are two major pathogenesis determinants found in all *S. enterica* serovars, encoding type III secretion systems (T3SS). Salmonella pathogenicity island 1 (SPI-1) is essential for colonization and invasion. SPI-1 encodes several transcriptional regulators, including HilA, HilC, HilD, and InvF.

Bile has different regulatory effects on the SPI-1 T3SS in different species. For instance, bile suppresses the expression and activity of the *S.* Typhimurium SPI-1 T3SS while increasing the SPI-1 T3SS of *S. typhi*. *Salmonella typhi* accomplishes this by extending the half-life of HilD and raising the expression of SipC, SipD (translocon protein), and SopB, SopE (Salmonella outer protein) [32].

### 4.2. Non-Coding RNAs

Non-coding RNAs are a type of regulatory RNA that is not translated into proteins but plays a transcriptional and post-transcriptional regulatory role in gene expression. Authors, such as Chen et al., have discovered a novel cis-encoded lncRNA AsfD encoded by the antisense strand of the flhDC operon [54]. They observed that AsfD is associated with *S. typhi* biofilm development and the transcription of AsfD is induced by the regulators OmpR and Fis. AsfD positively regulate *S. typhi* motility as well as biofilm formation by up-regulating different flagellar genes and the target gene of flhDC operon [54]. Another novel non-coding RNA (ncRNA), RibS, was found to be associated with biofilm formation in *S. typhi*. The *RibS* promote *S. typhi* biofilm formation by increasing the expression of the cyclopropane fatty acids synthase gene (*cfa*), which encodes cyclopropane fatty acid (CFA) synthase and catalyzes the conversion from unsaturated fatty acids to CFAs [55,56]. The cyclopropane fatty acid synthase is abundant in the shear removable section of the biofilm; an increase in CFA content in the cell wall or extracellular matrix might be beneficial to the production of bacterial biofilms.

### 4.3. Plasmid Containing Genes Related to Biofilm

The 1980s saw a large outbreak of *Salmonella enterica* serovar Typhi. Five hundred and ninety-one strains were obtained from individuals with acute and severe clinical symptoms. More than 80% of isolates were multi-drug resistant, the consequence of which was identified as pRST98. pRST98 is a large chimerical plasmid that contains the Salmonella plasmid virulence gene- spv involved in drug resistance and virulence [57]. Bacteria containing pRST98 produced sticky, viscous pellicles, whereas bacteria without pRST98 formed looser, less coherent biofilm [58]. It is also reported that pRST98 enhanced Salmonella serum resistance and improved S. *typhi* survival in macrophages in vitro; pRST98 inhibited autophagy in macrophages, therefore, impairing host cells’ innate immunity [59,60,61].

*Salmonella enterica* serovars Enteritidis and Typhimurium have the rck gene on their virulence plasmids. It was discovered that the rck gene is located on pRST98 [57]. By activating rck transcription, pRST98 enhanced the cellular adhesion of bacteria and increased bacterial resistance to serum. Thus, it has been postulated that rck may regulate biofilm developments in *S. typhi*. However, conjugative plasmids have a complex mechanism for influencing the formation of biofilm. Therefore, this rck-pRST98-mediated biofilm production for *S. typhi* requires more research.

### 4.4. Genes Related to Biofilm

Mig-14 is an inner-membrane-associated protein. Mig-14 and virK genes facilitate *Salmonella enterica* resistance to a variety of antimicrobial peptides, including polymyxin B (PB) [62]. Many mobile genetic elements such as Mig-14 are a common motif in Salmonella pathogenesis, and they can boost the bacterium’s virulence by searching out a new host or enhancing their own resistance [63]. However, the method through which Salmonella resists PB by Mig-14 is currently unclear. Recently, it was shown that Mig-14 plays a significant role in *S. typhi* resistance to PB by lowering the permeability of the outer membrane and encouraging the growth of biofilms [64]. Mig-14 is unable to change the structures of lipopolysaccharide (LPS) itself in the presence of PB. However, some adverse environments, such as antimicrobial peptides and acidic pH conditions, may stimulate Mig-14 expression, showing that the global regulator PhoP is in charge [65]. PhoP is one of the two key regulators that Salmonella virulence genes require to survive in macrophages [64]. Thus, it can be clear that Mig-14 is crucially connected with biofilm formation within the bile-acid-rich gallbladder, even after antimicrobial treatment. Researchers have found evidence that the outer membrane of *S. typhi* OmpF and OmpC has a significant role in antibiotic resistance by altering the outer membrane’s permeability, and Mig-14 was discovered to contribute to PB resistance by lowering the expression of OmpF and OmpC [64].

GalE has been found to be essential for developing biofilms in their final phases. It is a protein-coding gene that synthesizes galactose, which is used to make the outer core and the O-antigen of Salmonella lipopolysaccharides [45]. GalE has also been found to have a role in the production of the sugars needed to generate colanic acid, an EPS component in *E. coli* that has been linked to biofilm development [66,67]. GalE usually encodes a uridine diphosphogalactose-4-epimerase; however, a mutation in this gene results in a rough or incomplete LPS. To examine that hypothesis, Tn10 (a transposable element) was inserted in GalE to develop a biofilm on gallstones. Results indicated that the GalE mutant could develop a biofilm after fourteen days, but had fewer web-like strands and was much thinner than the wild-type strands [45].

### 4.5. Role of Quorum Sensing (QS) in Biofilm Formation

Bacterial cells have mechanisms that help them to cooperate in stressful situations and quorum sensing is one of the major mechanisms. It is one kind of bacterial communication system, and has been implicated in the biofilm formation of many bacteria. QS is also believed to influence the virulence of both typhoidal and non-typhoidal Salmonella strains [68]. *S. typhi* use quorum sensing to communicate cell–cell signals and coordinate gene expression [69]. The quorum sensing mechanism is mediated by three types of autoinducers (AI), autoinducer I, II, III, but autoinducer II and III are important regulators of pathogenic molecules in Salmonella [70,71,72].The autoinducer II may assist bacteria in transition from a pathogenic to a free-living condition in the environment [41]. Salmonella enterica produce autoinducer II (AI-2) through the luxS synthase gene, which is used by some bacterial pathogens to control the expression of virulence genes. To demonstrate that, a mutant deletion of the luxS gene was constructed and grown in bile-rich media. After 16 h of incubation in the presence of bile, the luxS::Km (the *S*. *typhi* mutant lacking the ability to produce the quorum-sensing signal molecule AI-2) strain’s biofilm-forming capacity was shown to be reduced when compared with control strain. Additionally, when exposed to bile, the mutant (luxS::Km) exhibited much lower levels of SOD and catalase than the control strain. Moreover, when compared with the control, luxS::Km reported a 40% growth inhibition in response to bile stress [29]. The deletion of the *luxS* gene resulted in the down-regulation of motility-related genes, salmonella pathogenicity island genes, and chemotaxis genes [73]. Furthermore, the interruption of the luxS coding sequence might indicate interference with MicA production, a short non-coding RNA molecule required for optimal biofilm development in Salmonella [74].

N-acylhomoserine lactones (AHLs) are quorum-sensing (QS) signaling molecules that respond to bacterial population density and activate various gene expressions. However, AHLs are not produced in Salmonella. When other bacteria generate AHLs, Salmonella produces a receptor SdiA that reacts to those AHLs [75]. However, more research is needed to screen more effective quorum-sensing compounds to control *S. typhi* biofilm formation.

### 4.6. QseB- and QseC-Mediated Biofilm Formation

The QseBC two-component system (TCS) acts as a universal regulator of virulence genes associated with quorum sensing, whereas CQseB acts as a response regulator for these two component systems [76,77]. Researchers found that the biofilm formation ability of the QseB and QseC mutant (lack of QseB and QseC) is much lower than the control. They have hypothesized that QseB in *S. typhi* may have two distinct functions in the regulation of biofilm-related genes, one of which is determined by its phosphorylation state in a QseC-dependent manner. In the presence of QseC, QseB aids the growth of biofilms. Meanwhile, in the absence of QseC, QseB is activated in an unusual way and plays an adverse effect on biofilm formation [78]. However, abnormal QseB activation has a negative impact on epithelial cell invasion in the absence of QseC, which has a significant effect on the biofilm [78].

**Table 1:** Function of different regulatory molecules in biofilm formation of *Salmonella typhi*.

Biofilm-Related Regulatory Molecules in *S. typhi*	Function	References
lncRNA *AsfD*	Increase S. *typhi* motility by up-regulating different flagellar genes	[51]
ncRNA *Ribs*	Up-regulates the expression of cyclopropane fatty acids synthase gene (cfa) that promotes biofilm	[53]
*Mig-14* and *Virk* genes	Decrease the permeability of the outer membrane for PB and encourage the growth of biofilms.	[61]
*GalE* gene	Synthesize galactose which are added to the outer core and the O-antigen	[41]
*Rck* gene	Enhance cellular adhesion of bacteria and promote biofilm formation.	[54]
*LuxS* gene	Encodes autoinducer 2 (AI-2), an essential part of the quorum-sensing mechanism.	[27]
pR ST98 plasmid	Contains genes that may be related to biofilm formation.	[55]
QseB and QseC	Involved in motility and biofilm formation.	[75]

## 5. Conclusions

As a key component of Salmonella’s virulence, biofilm has gained substantial interest from researchers. A growing number of studies on biofilms have revealed that it is far more complicated than previously thought. Since *S. typhi* infects only humans, we have a limited comprehension of the disease pathogenesis. Numerous research projects have relied on murine models using *S. enterica* serovar Typhimurium, which causes a typhoid-like disease in mice. Thus, most of what we know about *typhi* pathogenicity comes from studies on Typhimurium infections in mice. Although the pathogenicity of *typhi* and Typhimurium is vastly different, they share a large number of identical genes for biofilm formation. However, several genes that were found in Typhimurium haven’t been looked at in *S. typhi*. Nowadays, Salmonella’s growing antibiotic resistance poses a public health risk that is primarily dependent on biofilm formation in the gallbladder. Screening for biofilm-forming *S. typhi* early in infection is strongly advised for an improved therapeutic approach and antibiotic selection. This review, therefore, will put a spotlight on factors involving *S. typhi* biofilm formation and its long-term pathogenicity inside the human body.

## Data Availability

Not applicable.
